# Positive predictive value highlights four novel candidates for actionable genetic screening from analysis of 220,000 clinicogenomic records

**DOI:** 10.1038/s41436-021-01293-9

**Published:** 2021-08-13

**Authors:** Kelly M. Schiabor Barrett, Alexandre Bolze, Yunyun Ni, Simon White, Magnus Isaksson, Lavania Sharma, Elissa Levin, William Lee, Joseph J. Grzymski, James T. Lu, Nicole L. Washington, Elizabeth T. Cirulli

**Affiliations:** 1 Helix, San Mateo, CA USA; 2 Renown Institute for Health Innovation, Reno, NV USA; 3 Desert Research Institute, Reno, NV USA

## Abstract

**Purpose:**

To identify conditions that are candidates for population genetic screening based on population prevalence, penetrance of rare variants, and actionability.

**Methods:**

We analyzed exome and medical record data from >220,000 participants across two large population health cohorts with different demographics. We performed a gene-based collapsing analysis of rare variants to identify genes significantly associated with disease status.

**Results:**

We identify 74 statistically significant gene–disease associations across 27 genes. Seven of these conditions have a positive predictive value (PPV) of at least 30% in both cohorts. Three are already used in population screening programs (*BRCA1*, *BRCA2*, *LDLR*), and we also identify four new candidates for population screening: *GCK* with diabetes mellitus, *HBB* with β-thalassemia minor and intermedia, *PKD1* with cystic kidney disease, and *MIP* with cataracts. Importantly, the associations are actionable in that early genetic screening of each of these conditions is expected to improve outcomes.

**Conclusion:**

We identify seven genetic conditions where rare variation appears appropriate to assess in population screening, four of which are not yet used in screening programs. The addition of *GCK*, *HBB*, *PKD1*, and *MIP* rare variants into genetic screening programs would reach an additional 0.21% of participants with actionable disease risk, depending on the population.

## INTRODUCTION

Genetic conditions that are appropriate for population screening in US health programs are recommended to meet multiple criteria as proposed in guidelines by the CDC and/or American College of Medical Genetics and Genomics (ACMG) [1, 2]. Broadly, they must be conditions that affect a large number of people, have a genetic component with high penetrance in unselected populations, benefit from identifying at-risk individuals before they have fully developed the condition, have clear actionability for a change in clinical care upon genetic identification, and have the utility of screening confirmed by appropriate health economic analyses. An example of such conditions includes the CDC Tier 1 conditions—*BRCA*-related hereditary breast and ovarian cancer (HBOC), Lynch syndrome (LS), and familial hypercholesterolemia (FH) (Table 1)—which have highly penetrant and actionable genetic associations [1]. In contrast, the ACMG has identified 73 genes recommended for return of results of secondary findings, but most are not currently recommended for population screening because, although they have many of the same properties as CDC Tier 1, they are often too rare to be identified in population studies and have not undergone thorough analyses of their clinical and economic impact [3].

**Table 1:** Positive predictive value (PPV) estimates from population-level genetic screening programs in health systems.

**Genetic condition included in population screening**			**PPV of heterozygous variants in unselected cohorts: personal history (plus family history)**			
Condition name	Primary diseases	Genes	Geisinger MyCode^4^	UK Biobank^5c^	Mount Sinai BioMe^6^	Healthy Nevada Project ^7c^
Hereditary breast and ovarian cancer (HBOC)	Breast and ovarian cancers in females	*BRCA1*, *BRCA2*^a^	15%^﻿b^ (48%)	28% (30%)	37% (57%)	32% (NA)
Familial hypercholesterolemia (FH)	Atherosclerotic cardiovascular disease	*LDLR*, *APOB*, *PCSK9*	NA^d^	21% (65%)	NA	18% (NA)
Lynch syndrome	Colorectal and uterine cancers	*MLH1*, *MSH2*, *MSH6*, *PMS2*, *EPCAM*	32% (60%)	22% (39%)	NA	29% (NA)

^a^Additional genes associated with hereditary breast cancer not examined in the studies shown here include *ATM*, *PALB2*, *CHEK2*, *TP53*, *PTEN*, and *CDH1*.

^b^Does not appear to be limited to females.

^c^The UK Biobank and Healthy Nevada Project cohorts referenced here overlap with the samples used in the present analysis, but the sample sizes are larger now, and the definition of likely disease-causing variants is different.

^d^Does not report an atherosclerotic cardiovascular disease phenotype, but does report 96% PPV for elevated LDL-C.

In health systems currently offering population genetic screening based on CDC Tier 1 conditions, roughly 1% of an unselected patient population harbors a pathogenic/likely pathogenic (P/LP) variant, and as many as 80% of these individuals are unaware of their elevated risk status [4, 5]. Leveraging available health-care data, individuals with P/LP variants as a group display roughly 2–40 times higher risk of developing disease as compared to those without variants, and they also demonstrate penetrance averaging between 20% and 35% for personal history of relevant disease, increasing to 30–65% when family history is also considered, which helps contextualize lifetime risk of disease development (Table 1) [4–7]. This means that there is a high positive predictive value (PPV), generally >30%, when identifying individuals with P/LP variants.

Given the real world prevalence and penetrance seen thus far in genetic screening programs that detect and report P/LP variants, identifying additional common diseases where genetic variants confer a high PPV would expand the benefits of genomic medicine and population screening, as well as improve our understanding of disease biology.

In our opinion, the best candidates to expand genetic screening programs are those rare variants that predispose individuals to common diseases. Compared to common variants, rare variant associations are much more penetrant, resulting in direct and often more severe phenotypic effects that are also often relevant across ethnicities [8]. Significant rare variant associations at the population level not only distinguish differences in relative risk of disease between individuals with rare variants and control groups (often quantified as an odds ratio or OR), but also have high PPV, indicating a high probability for individuals with the variant to develop the disease in question. The high PPVs seen with many associated rare variants are similar to relationships established for known P/LP variants. These results thus have both high clinical validity and high clinical utility when used prospectively to modify disease outcomes. When individuals with variants are identified prior to disease onset, proactive actions such as diagnostics, monitoring, and prophylactic risk reducing procedures, often beyond or different from the standard of care, can be employed to prevent or modify the disease for these individuals.

Because of this high PPV, the prospects of larger or additional cohorts for rare variant analyses are very different from potential benefits of larger sample sizes in common variant association analyses. While larger sample sizes in studies of common variants identify signals with smaller and smaller effect sizes, larger sample sizes in studies of rare variants allow for the identification of rare causal variants that can be used to very precisely inform an individual about their risk of disease. Here, we leverage exome and medical data from two large health-care cohorts to identify rare variant—common disease relationships that are statistically significant at the population level, with high PPV (≥30%) and actionability relevant to the individuals with the variants, in line with the recommendations for population screening programs.

## MATERIALS AND METHODS

### Study design

As prior studies have shown, reducing the dimensionality of the genetic inputs can improve the power to detect associations with phenotypes when analyzing rare variants at the population level [9, 10]. Furthermore, differences in billing code (ICD) practices can artificially dampen diagnosis phenotype resolution both within and across cohorts and, like genetic signals from rare variants, they may also benefit from grouping methods [11]. Here, we performed genetic disease association analyses with two large exome-sequenced cohorts, the UK Biobank (UKB, *n* = 189,495) and Healthy Nevada Project (HNP, *n* = 28,423), using both gene and phenotype collapsing techniques.

### Populations and genetic data

We utilized the OQFE version of the UKB PLINK-formatted exome files (field 23155) as well as the imputed genotypes from genome-wide association study (GWAS) genotyping (field 22801–22823). The HNP samples were sequenced and analyzed at Helix using the Exome+^®^ assay as previously described [9]. The UKB participants range in age from 40 to 69 and are 55% female, while the HNP age range is from 18 to 89+ and is 68% female. The UKB is 83% British European ancestry, with another 10% of other European ancestry and 7% other ancestries, and the HNP is 77% general European ancestry, 14% Hispanic ancestry, and 9% other ancestries.

### Phenotypes

HNP phenotypes were processed from Epic/Clarity Electronic Health Records (EHR) data as previously described [9]. UKB data were provided from the UKB resource (http://www.ukbiobank.ac.uk/, accessed August 2020). For HNP, International Classification of Diseases, Ninth and Tenth Revision ICD codes (ICD-9 and ICD-10-cm) were collected from available diagnosis tables (from problem lists, medical histories, admissions data, surgical case data, account data, claims, and invoices). For UKB, ICD codes (both ICD-9 and ICD-10) were collected from inpatient data, cancer registry table, and the first occurrences table (resource 593).

To map ICD to phecodes, ICD-9 (Phecode Map 1.2, used for both cohorts), ICD-10 (Phecode Map 1.2b to ICD-10 beta, used for UKB), and ICD-10-CM (Phecode Map 1.2b to ICD-10-CM beta, used for HNP) to phecode maps from the Phewas catalog were used to code individuals as a 1 if they had the phecode recorded at least once in their medical records, and otherwise 0 [12–14]. Analysis phenotypes were restricted to have cases in both cohorts, with at least 30 cases in the HNP data set (*n* = 1,044 phenotypes).

When identifying age at diagnosis, we required at least 5 years of medical history prior to the diagnosis, meaning the first diagnosis of any condition in the record must occur at least 5 years prior to the diagnosis in question, except for when diagnosis occurred in the first five years of life.

### Gene-based collapsing

Variant annotation was performed with VEP 99 [15]. Coding regions were defined according to Gencode version GENCODE 33, and the Ensembl canonical transcript was used to determine variant consequence [16, 17]. Variants were restricted to CDS regions. Genotype processing was performed in Hail 0.2.54-8526838bf99f.

For the collapsing analysis, samples were coded as a 1 for each gene if they had a qualifying variant and a 0 otherwise [9]. We defined “qualifying” as coding (stop_lost, missense_variant, start_lost, splice_donor_variant, inframe_deletion, frameshift_variant, splice_acceptor_variant, stop_gained, or inframe_insertion) and not PolyPhen or SIFT benign (PolyPhen benign is <0.15, SIFT benign is >0.05). We also ran a loss-of-function (LoF) model that only included LoF variants (stop_lost, start_lost, splice_donor_variant, frameshift_variant, splice_acceptor_variant, or stop_gained). Variants were only included if their minor allele frequency (MAF) was below 0.1% in all gnomAD populations as well as locally within each population analyzed. Only variants that passed our MAF and predicted function thresholds were included, regardless of known P/LP status.

### CNVs calls in HNP data

The Helix Exome+^®^ platform includes a copy-number variant (CNV) caller, allowing us to incorporate rare CNVs at exon-level resolution into our gene-based collapsing analysis for the HNP samples [18]. Briefly, CNVs with the PASS QC filter were annotated with overlapping canonical transcripts (CT). For the collapsing analysis, rare CNV events were screened using both exon and event-level frequency information from within the cohort (<0.1% for each), as well as by relevant CNV type—deletions of at least one exon of the gene for LoF model, and deletions or duplications for damaging. Information on how many individuals carried CNVs in each significantly associated gene can be found in Table S1. Including CNVs increased the median frequency of individuals with variants in each gene by ~8%.

### Genetic analysis

We used regenie for the genetic analysis [19]. Briefly, this method builds a whole-genome regression model using common variants to account for the effects of relatedness and population stratification, and it accounts for situations where there is an extreme case–control imbalance, which can lead to test statistic inflation with other analysis methods. The covariates we included were age, sex, age*sex, age*age, sex*age*age, and bioinformatics pipeline version as appropriate.

As previously described, a representative set of 184,445 coding and noncoding linkage disequilibrium (LD)-pruned, high-quality common variants were identified for both the creation of principal components and for building the whole-genome regression model [9].

We performed two main analyses: (1) all ancestries together and (2) only European ancestry, with 10 European ancestry-specific principal components included as additional covariates. When collapsing rare (MAF <0.1%) causal variants across a gene and analyzing with a linear mixed model or whole-genome regression, signals tend to be consistent whether restricting to one ancestry or analyzing across all ancestries [9]. This method works in this setting because analyses of collapsed rare variants are less influenced by ethnic background than are analyses of the common variants used in a typical GWAS, in large part because causal variants are being grouped together as opposed to tagging variants.

Meta-analysis was performed using the weighted *Z*-score *p* value in METAL [20] on the summary stats from each separate analysis. QQ plots showed no test statistic inflation. We required at least one individual to have the variant in both the UKB and the HNP groups, and the meta *p* value to be lower (better) than the *p* values for either individual cohort.

To identify significant associations, we used a conservative Bonferroni correction for multiple tests for all genes that had individuals with qualifying variants (*p* < 1 ×10^-9^).

### PPV cutoff

To classify gene–disease relationships that would be strong candidates for population screening, we first calculated the PPV (percent of individuals with the variant who develop the condition) of each significant gene-based association by grouping individuals based on age, either all ages (ages 18–89+) or only 60+, to better estimate lifetime risk. Based on the PPV of genetic conditions typically reported in existing genetic screening programs (Table 1), we selected a PPV threshold of ≥0.3 to partition our association results. We applied this threshold to both the all ages and lifetime risk groups, and we included those associations from the 60+ group even if the PPV was lower prior to age 60.

## Results

### Population-level associations

Our gene-based collapsing analysis of rare variants included 15,857 genes in the coding model, 15,617 of which were also in the LoF model. For the phenotypes, we used phecodes to reduce the phenotype complexity from >20,000 ICD 9 and 10 codes to simply 1,044 medically relevant phenotypes based on available electronic health records (EHR) for both HNP and UKB cohorts. Our meta analysis across both data sets identified 74 statistically significant associations (*p* < 1×10^-9^) between 27 genes and 49 phecodes (Table 2 and Table S1). While most of the significant associations were obtained with a LoF model, 29 were associations found with coding models, including eight genes for which there was no significant LoF association (the association was only with the coding model).

**Table 2:** Population-level significant rare variant gene–disease (*p* < 1x10^−9^) associations.

**Gene**	**Model**	**Phenotype**	***P*** **value**	**OR**	**PPV ≥0.3 in both cohorts**	
					**Age 60+**	**All ages**
*HBB*	LoF	Other hemoglobinopathies	1.91E-129	197.2	+	+
*PKD1*	LoF	Cystic kidney disease	4.54E-48	78.5	^b^	+
*GCK*	Coding	Type 2 diabetes	1.46E-33	11.3	+	+
*LDLR*	LoF	Coronary atherosclerosis^a^	1.46E-12	17.5	+	+
*BRCA2*	LoF	Malignant neoplasm of female breast^a^	3.96E-45	8.5	+	-
*BRCA1*	LoF	Malignant neoplasm of female breast^a^	8.77E-28	14.2	+	-
*MIP*	Coding	Cataract	1.56E-10	4.6	+	-
*JAK2*	Coding	Myeloproliferative disease^a^	6.41E-62	7.6	-	-
*COL4A4*	LoF	Hematuria	8.96E-23	4.6	-	-
*TTN*	LoF	Atrial fibrillation and flutter^a^	1.91E-17	1.8	-	-
*MSH6*	LoF	Malignant neoplasm of uterus	2.11E-17	19.6	-	-
*MYBPC3*	LoF	Other hypertrophic cardiomyopathy	5.07E-17	70.2	-	-
*IFT140*	LoF	Cyst of kidney, acquired	3.81E-16	10.2	-	-
*NF1*	LoF	Other benign neoplasm of connective and other soft tissue	1.25E-15	14.9	-	-
*PKD2*	Coding	Cystic kidney disease	1.85E-15	3.9	-	-
*TET2*	LoF	Neutropenia^a^	2.34E-15	4.8	-	-
*VWF*	Coding	Von Willebrand disease	2.77E-15	6.7	-	-
*SF3B1*	Coding	Myeloproliferative disease	4.21E-13	13	-	-
*CDKN2A*	Coding	Melanomas of skin	5.57E-13	10.2	-	-
*TSHR*	Coding	Hypothyroidism not otherwise specified	1.52E-12	1.9	-	-
*PALB2*	LoF	Malignant neoplasm of female breast	8.22E-12	5.0	-	-
*ASXL1*	LoF	Myeloproliferative disease	9.75E-12	13.0	-	-
*PROC*	Coding	Phlebitis and thrombophlebitis^a^	1.35E-11	4.9	-	-
*ATM*	LoF	Malignant neoplasm of female breast	2.49E-11	4.9	-	-
*SLC22A12*	Coding	Gout	4.86E-11	0.1	-	-
*MLH1*	LoF	Colon cancer	1.54E-10	240.7	-	-
*SLC4A1*	Coding	Other hereditary hemolytic anemias	1.99E-10	19.8	-	-

*LoF* loss of function, *OR* odds ratio, *PPV* positive predictive value.

^a^A significant association was also found with another phenotype with a PPV that was higher than that for the main phenotype, but it was not a clinical endpoint of main interest (for example, acquired absence of breast for *BRCA1/2*, or hypercholesterolemia for *LDLR*). For full details, see Table S1. ^b^The PPV was >0.3 at age 60+ in UKB, but all 6 HNP *PKD1* LoF heterozygotes with cystic kidney disease were aged <60.

Importantly, the ethnic makeup of the two cohorts was quite different despite each being predominantly of European ancestry, and our analysis results were similar whether restricting to European ancestry or analyzing across ethnicities (Table S1), consistent with our previous study showing that collapsed rare variant signals tend to be consistent across ancestries [9].

### Applying PPV to highlight associations for population genetic screening

**Fig. 1:**
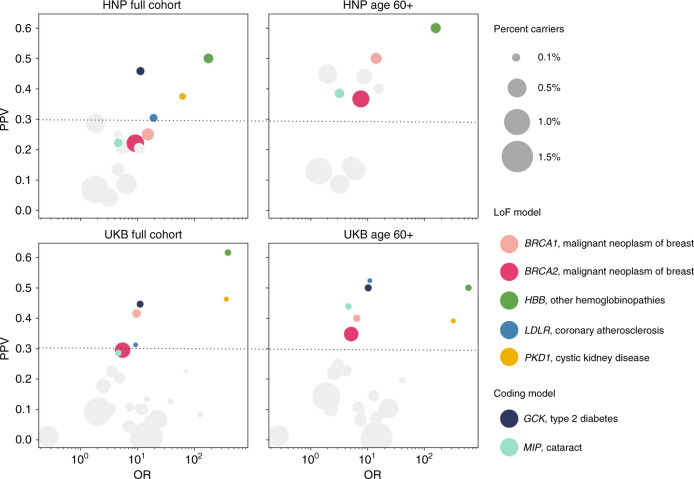
Positive predictive value (PPV) vs. odds ratio (OR) for statistically significant associations. Shown is the significant association with the best PPV for each gene, as in Tables 2 and 3. The horizontal line indicates our PPV cutoff of 0.3 for high impact genes. The percent of the cohort with variants of interest for each gene is shown by the size of the circle. The seven genes with PPVs ≥0.3 in both cohorts are shown in colors as indicated in the legend, and the remaining genes are in gray. Because low sample sizes can produce unreliable results, only data points with at least five cases with variants are shown (this excludes *PKD1*, *GCK*, and *LDLR* in the HNP age 60+ subset). The three gene associations that are above the 0.3 cutoff in HNP age 60+ but had lower PPVs in UKB are detailed in Tables 2 and S1 and include *IFT140* with acquired cyst of kidney, *TSHR* with hypothyroidism, and *ATM* with malignant neoplasm of breast.

We identified seven genes that passed our PPV cutoff of 0.3 (meaning at least 30% of individuals who carried qualifying variants developed the condition). It is important to note that we required the PPV to be above this threshold for *both* cohorts, indicating that the predictive power of the genetic association is applicable across different health systems, population demographics, and countries. Additionally, the ORs for these associations were all >4 in both cohorts, indicating a substantial increase in risk. As expected, some of the statistically significant associations that meet or exceed this threshold cover gene–disease relationships that are already tested in existing population screening programs: *BRCA1* and *BRCA2* with breast cancer (*BRCA1* *p* = 8.77×10^-28^, OR = 14.2; *BRCA2* *p* = 3.96×10^-45^, OR = 8.5), and *LDLR* with coronary atherosclerosis (*p*  = 1.46×10^-12^, OR = 17.5). Additionally, we observed several statistically significant associations that have just as strong or stronger PPVs than these conditions, including LoF variants in *HBB* with hemoglobinopathies (*p*  = 1.91×10^-129^, OR = 197.2), LoF variants in *PKD1* and with cystic kidney disease (*p* = 4.54×10^-48^, OR = 78.5), coding variants in *GCK* with diabetes mellitus (*p* = 1.46×10^-33^, OR = 11.3), and coding variants in *MIP* with cataracts (*p* = 1.56×10^-10^, OR = 4.6) (Table 2 and Fig. 1). The remaining significant associations have PPV <0.3 and would have more limited utility if communicated to patients under this paradigm (Table 2 and Table S1**)**.

Importantly, each high-PPV gene–disease association identified here is actionable at some level, further supporting their suitability for inclusion in population screening programs. While some of the conditions have clearly established preventive guidelines based on genetics, all would benefit from earlier diagnosis. Since genetic screening for highly penetrant conditions can lead to a more accurate diagnosis, the resulting medical management guidelines for the patients are likely to be improved. For example, treatment recommendations for maturity onset diabetes of the young (MODY) vary depending on the genetic status of the patient. Individuals who have a *GCK* variant generally do not need treatment and can benefit from a reduced need for surveillance so long as any hyperglycemia remains the mild fasting hyperglycemia typically seen with *GCK*. Clinical actionability, medical management, surveillance methods, and genetics-dependent care pathways are summarized for these associations in Table 3 and discussed further below.

**Table 3:** Summary of PPV and clinical actionability for genes with significant associations and PPV ≥0.3 in our study.

**Condition**	**Hereditary breast and ovarian cancer**^**a**^		**Type 2 diabetes**^**a**^	**Gestational diabetes**	**β-thalassemia minor and intermedia**^**b**^	**Familial hypercholesterolemia**^**a**^	**Cataracts**	**Chronic kidney disease**
Associated complications	Breast, ovarian, prostate, and pancreatic cancers and melanoma		Microvascular disorders	Macrosomia	Anemia, osteoporosis, iron overload	Atherosclerotic cardiovascular disease	Blurry vision up to blindness	Early onset hypertension, cyst infections
Nongenetic surveillance available to confirm or monitor disease?	Mammograms, MRIs, transvaginal ultrasound, regular CA-125 surveillance, PSA surveillance		Glucose and HbA1C surveillance	OGTT	CBC with smear, hemoglobin analysis	Lipid blood tests	Slit lamp eye exam	TKV, serum creatinine, eGFR
Treatment options (to mitigate, prevent, or reverse)	Risk-reducing surgeries		Diet, exercise, metformin, other oral hypoglycemic agents, insulin	Diet change and intense monitoring; insulin needed if diet and exercise do not help	Supplementation, blood transfusion, iron chelation	Statins. May also consider ezetimibe, bile acid sequestrants, niacin, *PCSK9* inhibitors, LDL apheresis	Cataract surgery	Various drug options to slow progression
Associated gene	*BRCA1*	*BRCA2*	*GCK*		*HBB*	*LDLR*	*MIP*	*PKD1*
% with variant^c^	0.11%	0.36%	0.06%		0.06%^c^	0.03%	0.06%	0.03%
PPV^c^	0.43	0.35	0.5	0.82^e^	0.59	0.5	0.43	0.44
Diagnose earlier with genetic screening, and cascade test?	Yes	Yes	Yes	Yes	Yes^f^	Yes	Yes	Yes
How are genetic cases treated differently?	Earlier monitoring/treatment	Earlier monitoring/treatment	*GCK* forms usually need no interventions [22]	Tailor treatment based on genotype of fetus	Usually do not give iron for anemia; reproductive counseling	Earlier monitoring/treatment	Earlier monitoring/treatment	Screen TKV to track disease progression [26]

*CBC* complete blood count, *eGFR* estimated glomerular filtration rate, *MRI* magnetic resonance image, *OGTT* oral glucose tolerance test, *PPV* positive predictive value, *TKV* total kidney volume.

^a^Conditions already part of existing population screening programs and part of CDC Tier 1.

^b^Complications and treatments differ depending on the exact type of β-thalassemia, with β-thalassemia minor having no complications beyond mild anemia.

^c^Frequency varies substantially by population.

^d^Calculated for the significant phenotype and model with the highest PPV for this gene in this study.

^e^When controls are restricted to pregnant females.

^f^While symptomatic thalassemia is generally diagnosed in childhood, 71% of the heterozygotes in the present study were diagnosed as adults.

Overall, we find seven associations with high PPV, four of which would be novel for population screening and warrant examination in additional cohorts to quantify suitability of screening in more genetically diverse populations, how well population screening can catch the conditions early and change disease course, and the resulting economic impact.

## DISCUSSION

Genetic screening programs that prospectively identify individuals who are likely to develop conditions that are treatable or preventable through medical interventions, especially when detected before disease onset or early in the disease course, could make substantial improvements to individual and public health. Rare variants that can be identified as causing common diseases in population-level analyses are the natural candidates for population screening programs due to their relatively high penetrance and prevalence. Here, we find that when conditions identified from gene-based collapsing analyses of rare variants consistently have a penetrance of at least 30% (PPV ≥0.3), they have properties that make them excellent candidates for population screening programs (Table 3). Our analysis identified seven such conditions. Four of these—coding variants in *GCK* with diabetes mellitus, LoF variants in *HBB* with hemoglobinopathies, LoF variants in *PKD1* with cystic kidney disease, and coding variants in *MIP* with cataracts—are novel conditions for population screening. It is notable that these four associations have a PPV as high or higher than the other three associations we identified, which are already used in population screening programs: LoF variants in *BRCA1* and *BRCA2* with HBOC and LoF variants in *LDLR* with atherosclerosis. These associations all represent genetically driven subsets of common, complex diseases that are in line with recommended guidelines for population screening and present opportunities for precision medicine at scale (Table 3) [2]. We briefly discuss each association below and the potential benefits of returning rare variant screening results to relevant individuals given current clinical knowledge and practice.

### *GCK* and type 2 diabetes

While often misclassified as type 2 diabetes (T2D), individuals with *GCK* variants typically have mild but stable fasting hyperglycemia and do not develop the microvascular complications typical of T2D [21]. The significant association (*p * = 1.46×10^-33^) and high PPV (0.5) we observe between *GCK* rare coding variants and T2D corroborates the misclassification of these cases seen in other studies, including ours [22]. Returning *GCK* results to relevant heterozygotes is actionable as it can help their health-care provider tailor the care they receive and set realistic goals for their glucose levels, which are unlikely to fall into the normal range regardless of lifestyle changes. With building evidence for no effect of oral or insulin treatment on glucose levels in *GCK* heterozygotes with mild hyperglycemia, identifying and terminating pharmaceutical treatments in these patients could lead to substantial lifestyle improvements and cost savings [23].

While *GCK* heterozygotes generally do not have problematic clinical outcomes for T2D, they are known to be at increased risk for developing gestational diabetes and are advised to be closely monitored during pregnancy [24]. Our analysis also identified a significant association between rare coding variants in *GCK* and gestational diabetes (Table S1), but the PPV did not pass our 0.3 cutoff (0.17 in HNP and 0.09 in UKB) because our main analysis for this trait included all females and was not restricted to pregnant females. However, when we limit our association analysis to include only females with pregnancy phenotypes in their medical records, we see the PPV for gestational diabetes rise to 1.0 for HNP and 0.75 for UKB (respectively, 0 of 2,363 and 2 of 10,555 pregnant females without gestational diabetes were heterozygous for qualifying *GCK* variants), suggesting this may indeed be a genetic condition worthy of prepregnancy population screening. In particular, identifying whether the fetus has inherited a *GCK* variant from either the mother or father can be important for tailoring care during pregnancy: in a pregnancy where the fetus has a *GCK* variant, hyperglycemia in the mother should usually not be treated as it can lead to dangerously low birthweight, while treatment with insulin is more likely to be indicated if the fetus did not inherit the *GCK* variant [24].

### *PKD1* and chronic kidney disease

Autosomal dominant polycystic kidney disease (ADPKD, caused by variants in *PKD1* and *PKD2*) is the most common inherited kidney disorder, is the fourth leading cause of chronic kidney disease, and is often not diagnosed until later stages of the disease [25]. While there is currently no cure for ADPKD, early detection of ADPKD can provide the opportunity to treat comorbidities such as early onset hypertension, cardiovascular complications, and cyst infections, and kidney disease progression can potentially be slowed with pharmaceutical intervention [26]. Genetic screening programs that include *PKD1* could help detect cases earlier and prioritize these patients for total kidney volume (TKV) measurements in addition to the more typical estimated glomerular filtration rate (eGFR) surveillance for better monitoring of disease progression.

In addition to the association seen with *PKD1*, we also saw a significant association between the related gene *PKD2* and cystic kidney disease (CKD) (Table 2). This coding model association had a lower PPV (OR = 12.5; PPV = 0.03), compared to that of the *PKD1* LoF model (OR = 292; PPV = 0.44). Further investigation of the data sets revealed that LoF variants in *PKD2* had a PPV of 0.5 in UKB (OR = 490; *p* value 2.5×10^-42^) but had not been included in the analysis because there were only 4 individuals with variants in total in HNP (OR~61; PPV = 0.5). Despite the similar effect sizes between LoF variants in *PKD1* and *PKD2*, LoF variants in *PKD2* occurred in only 0.02% and 0.01% of the UKB and HNP populations, respectively, compared to 0.03% and 0.06% for *PKD1*. With the HNP study continuing to enroll more participants, we will likely see additional individuals with a *PKD2* variant and CKD, which would likely revise this screening recommendation to include both *PKD1* and *PKD2* for CKD.

### *HBB* and hemoglobinopathies

Rare variants in *HBB* cause the recessive hemoglobinopathy β-thalassemia major, which is quite severe and presents early in life [27]. The statistically significant, dominant association between *HBB* rare variants and hemoglobinopathies and the high PPV (0.55, Table 3) found in our cohorts are driven by a mixture of some individuals who may have β-thalassemia intermedia, a less severe form of the disease that is sometimes inherited in a dominant fashion, and many individuals with β-thalassemia minor, who are generally asymptomatic but often have mild anemia [28, 29].

Individuals with β-thalassemia minor are often misdiagnosed as having iron deficiency anemia. In our study, 30% of *HBB* LoF heterozygotes with a thalassemia diagnosis and 16% of heterozygotes without a thalassemia diagnosis had a diagnosis of iron deficiency anemia, driving a statistically significant association with this trait (Table S1; compared to only 6% of those without a *HBB* LoF variant). Furthermore, 12% of *HBB* LoF heterozygotes reported taking iron supplements, compared to 3% of those without *HBB* LoF variants. Medical records indicated hemochromatosis in 1.6% of *HBB* LoF heterozygotes vs. 0.4% of those without *HBB* LoF variants, 2.4% vs. 0.007% had hepatic fibrosis, and 2.2% vs. 0.3% had nonalcoholic cirrhosis, indicating that complications of iron overload can be a concern for *HBB* LoF heterozygotes. Additionally, the bloodwork available for members of these cohorts showed that 100% of the *HBB* LoF heterozygotes, regardless of thalassemia diagnosis status, had red blood cell (RBC) microcytosis (mean corpuscular volume [MCV] <80 μm [3]; compared to 6% of those without LoF variants), indicating that many individuals with β-thalassemia minor may remain undiagnosed in these cohorts. For individuals with β-thalassemia intermedia, common complications include extensive iron overload in many tissues through increased intestinal absorption, as well as marked and progressive osteoporosis [27]. Not only can the diagnosis of thalassemia be directly confirmed via blood tests, but many screenings and treatments also exist to avoid or mitigate the phenotypic complications, including bone density scans, blood tests to assess iron overload, blood transfusions, splenectomy, folic acid supplementation, and iron chelation therapies [30, 31]. Early detection of *HBB* LoF heterozygotes is useful for reproductive planning and for helping physicians tailor treatment when considering the cause of the patient’s anemia. In our study, only 29% of cases with *HBB* LoF variants with age of diagnosis available had been diagnosed as children, indicating that genetic screening of adults for this condition may be warranted.

### *MIP* and cataract

While previous studies have implicated *MIP* variants in rare, familial, congenital cataracts, our results provide evidence for a more general role of *MIP* in cataracts [32–34]. The median age of cataract diagnosis in our study of adults was 61. Returning these genetic results at an earlier age provides an opportunity for health-care providers to encourage or even facilitate underutilized cataract screening and promote possible prevention strategies such as limiting UV exposure. The added risk may encourage yearly eye exams, as well as safe and effective routine surgery, for those at higher than average risk based on their genetics [35]. Cataract screening is typically performed as part of a routine eye exam, but relatively few Americans keep up with this practice. In a survey of the eye care usage trends of nearly 300,000 adults from 1997 to 2005, eye care utilization rates in the 12 months prior to survey for those older than 65, a group who not only receive coverage for an annual eye exam through Medicare but are also the most likely to harbor an eye condition like cataracts, ranged from 50% to 65% [35]. In addition to personal utility, the timely treatment of cataracts can also have societal benefits. Cataract surgery was recently associated with a 61% reduction in car crash frequency in a cohort of nearly 3,000 drivers aged 60 and above who underwent cataract surgery over the course of the study period [36]. On a broader scale, a deeper understanding of this genetic association has the potential to guide the development of pharmaceuticals that may slow or even reverse cataract disease progression [37, 38].

### Population-level clinical impact and future directions

When combining together the variant frequencies for all associations above our 0.3 PPV threshold, we find that population screening for these conditions could impact up to 1% of program participants (Table 3). Reassuringly, we identify genes (*BRCA1*, *BRCA2*, *and LDLR*) that are typically included in existing population health programs, which themselves account for more than half of the potential impact (0.47–0.73% of individuals have relevant variants in UKB and HNP, respectively). However, the inclusion of *HBB*, *GCK*, *PDK1*, and *MIP* in the same programs would reach an additional 0.19–0.36% of participants in each population (for UKB and HNP, respectively; this value will also differ by population, especially for *HBB*).

Recent economic evaluations have revealed that, in addition to personal utility, genetic screening programs are cost effective for payers, especially when performed earlier in life [39, 40]. Because all of the conditions identified here have evidence of improved outcomes when early actions are taken (Table 3), and given that there is a net increase in findings with the same amount of work at the population level (a single assay can just as easily screen one or all human genes), it is likely that the addition of these four conditions with the same or better PPV as existing population screening genes would only improve the cost effectiveness and overall economic benefit of a genetic screening program. However, additional work is still required by official clinical bodies to both evaluate the health economics of early intervention for these conditions and to translate these findings from research into clinical practice through official guidelines. In particular, guidelines will be needed to determine the type and frequency of screening modalities that will be needed for individuals who harbor risk alleles for these conditions. It is also important to include genetic counselors as a part of the return of results process and provide educational materials for all health-care providers involved in the communication of results. Therefore, the next step to expand the boundary of genomics in medicine is the creation, evaluation, and/or refinement of clinical guidelines based on genetics for these conditions.

## Supplementary information

Supplementary Materials

## Data Availability

UKB data are available for download (https://www.ukbiobank.ac.uk/). Analysis results for significant associations are available in Table S1. The HNP data are available to qualified researchers upon reasonable request and with permission of the Institute for Health Innovation (IHI) and Helix. Researchers who would like to obtain the raw genotype data related to this study will be presented with a data user agreement which requires that no participants will be re-identified and no data will be shared between individuals or uploaded onto public domains. The IHI encourages and collaborates with scientific researchers on an individual basis. Examples of restrictions that will be considered in requests to data access include but are not limited to (1) whether the request comes from an academic institution in good standing and will collaborate with our team to protect the privacy of the participants and the security of the data requested, (2) type and amount of data requested, (3) feasibility of the research suggested, (4) amount of resource allocation for the IHI and Renown Hospital required to support the collaboration. Any correspondence and data availability requests should be addressed to JG at (Joe.Grzymski@dri.edu) or Craig Kugler (Craig.Kugler@dri.edu).
